# Effects of exogenous melatonin on clinical and pathological features of a human thyroglobulin-induced experimental autoimmune thyroiditis mouse model

**DOI:** 10.1038/s41598-019-42442-0

**Published:** 2019-04-10

**Authors:** Jiunn-Diann Lin, Wen-Fang Fang, Kam-Tsun Tang, Chao-Wen Cheng

**Affiliations:** 1 Graduate Institute of Clinical Medicine, College of Medicine, Taipei Medical University, Taipei, Taiwan; 2 Division of Endocrinology, Department of Internal Medicine, Shuang Ho Hospital, Taipei Medical University, New Taipei City, Taiwan; 3 Division of Endocrinology and Metabolism, Department of Internal Medicine, School of Medicine, College of Medicine, Taipei Medical University, Taipei, Taiwan; 4 Department of Family Medicine, Shuang Ho Hospital, New Taipei City, Taiwan; 5 Division of Endocrinology and Metabolism, Department of Internal Medicine, Veterans General Hospital, Taipei, Taiwan; 6 Traditional Herb Medicine Research Center, Taipei Medical University Hospital, Taipei Medical University, Taipei, Taiwan; 7 Cell Physiology and Molecular Image Research Center, Wan Fang Hospital, Taipei Medical University, Taipei, Taiwan

**Keywords:** Autoimmunity, Thyroid diseases

## Abstract

Melatonin (MLT) plays a significant role in both innate and adaptive immunity, and dysregulation of the MLT signature can modify autoimmune disease phenotypes. In this study, the influence of exogenous MLT administration on regulating autoimmune thyroiditis animal models was evaluated. An experimental autoimmune thyroiditis model was established in MLT-synthesizing (CBA) and MLT-deficient (C57BL/6) mice by immunization with human thyroidglobulin (TG), which features thyrotoxicosis, thyrocyte damage, and CD3^+^ T cell infiltration. In TG-immunized CBA mice, exogenous MLT administration in drinking water (6 μg/ml) enhanced thyroiditis and increased TG-specific splenocyte proliferation but not the anti-thyroglobulin antibody (ATA) titer, while MLT alone caused no significant alteration in thyroid function or histopathology. Meanwhile, MLT administration did not modify thyroid function, the ATA titer, or the thyroid histopathology, but results showed an increase in the splenocyte proliferative capacity in TG-immunized C57BL/6 mice. Collectively, our data showed that early exogenous MLT modified the progression of autoimmune thyroiditis through T cell-driven immunity, and excess MLT worsened the clinical and pathological features.

## Introduction

Melatonin (MLT), is mainly synthesized by the pineal gland, and its secretion is inhibited by light to the retina but enhanced by darkness^1^. Circulating MLT binds to MT1 and MT2 membrane-bound receptors, and plays a role in regulating circadian and seasonal rhythms, and also works as a free radical scavenger, an antioxidant, and an anticancer adjuvant^2,3^. In addition, accumulating evidence has shown that the MLT signature acts as a modulator of both innate and acquired immune reactions, and an imbalance of the MLT signature can lead to dysregulated immunity^4–7^. Because of its prominent immunoregulatory activity, MLT has been extensively explored as a possible therapeutic strategy for multiple autoimmune diseases (AIDs), including systemic lupus erythematosus, multiple sclerosis, type 1 diabetes mellitus, and rheumatoid arthritis, but responses varied^8^.

The previous literature linked MLT to the thyroid gland in both *in vivo* and *in vitro* experiments, but the results remain controversial. Some studies showed that MLT could suppress the thyroid function in rats, while Gordon *et al*. reported that chronic MLT administration enhanced thyroid enlargement, increased radioiodine uptake (RAIU), and increased the total thyroid hormone amount without altering the thyroid hormone concentration in the bloodstream of rats^9–13^. Gesting *et al*. observed that MLT could inhibit aflatoxin-induced rat thyrocyte apoptosis, which suggested the potential role of MLT in controlling the growth of thyrocytes^14^. Wright *et al*. disclosed that thyroxine (T4) secretion was reduced in cultures of thyrocytes from different species incubated with MLT production compared to those in a control group^15,16^. Mogulkoc *et al*. also showed that MLT could prevent hyperthyroidism-induced tissue damage by reducing oxidative stress^17^. Interestingly, evidence demonstrated that MLT, together with its key synthesizing enzymes, and the MT1 receptor protein exist in the thyroid gland, which further implied that the MLT signature could exert certain biological activities in thyrocytes^18^.

Graves’ disease (GD) and Hashimoto’s thyroiditis (HT) are two extreme ends of autoimmune thyroid disease (AITD); in both of these diseases, specific thyroid T-lymphocytes are initially activated against a thyroid autoantigen, followed by induction of either B cell- or cytotoxic T cell-driven immunity, with an ultimate contribution to separate clinical and pathological features^19^. As aforementioned, because MLT exerts immunomodulatory properties and MLT and its key synthesizing enzymes are present in the thyroid gland, it was speculated that the MLT signaling pathway might also participate in the development of AITD or in modifying disease phenotypes. In our published genetic association study, we found that the single-nucleotide polymorphism of MTNR1A, coding the MT1 protein, was associated with a susceptibility to GD and thyroid autoantibody formation, which supports the notion that the MLT signature can influence the occurrence of AITD and clinical features^20^.

In this study, to further explore the effect of the MLT signature on the occurrence of AITD and modulation of disease phenotypes and progression, we utilized an experimental autoimmune thyroiditis (EAT) model by repeatedly injecting human thyroglobulin (TG) in two distinct mouse strains, CBA/CaJNarl (CBA) and C57BL/6J (B6), features with differences in the production of MLT. Subsequently, MLT was exogenously given to the mouse model, and we further evaluated the influence of MLT on the outcome of a TG-immunized mouse model and its potential immunological regulation.

## Results

### Exogenous MLT treatment enhances plasma T4 and triiodothyronine (T3) levels of TG-induced thyroiditis

**Figure 1:**
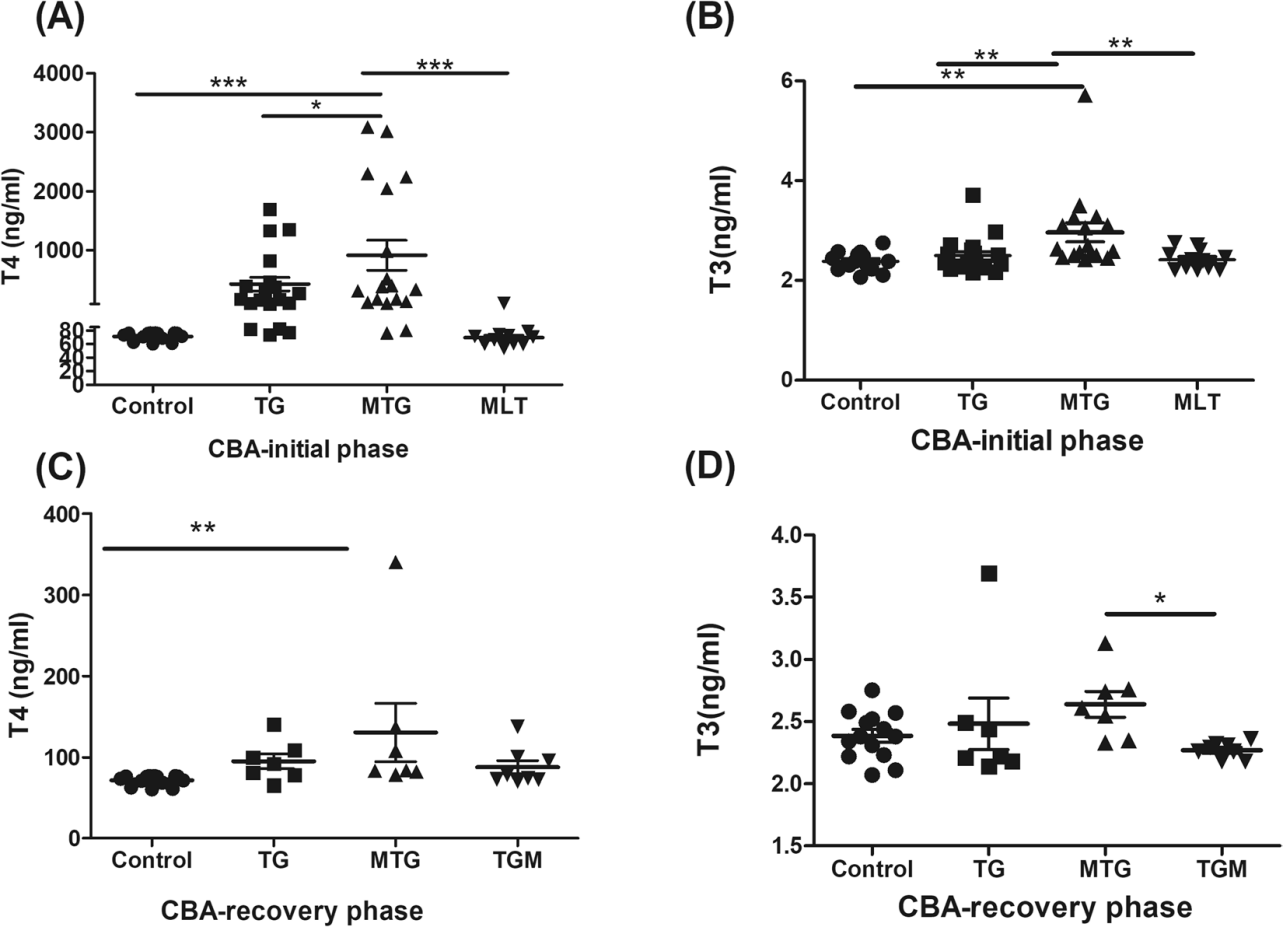
Thyroid functions in the different groups of CBA mice in the initial and recovery phases. (**A**) Plasma thyroxine (T4) levels in the different groups in the initial phase. (**B**) plasma triiodothyronine (T3) levels in the different groups in the initial phase. (**C**) T4 levels in the different groups in the recovery phase. (**D**) T3 levels in the different groups in the recovery phase. Each bar represents the mean and standard error. *p < 0.05, **p < 0.01; ***p < 0.001.

Repeated immunization with TG in CBA mice (TG group) led to the development of thyrotoxicosis, as manifested by increases in plasma T4 and T3 levels (Fig. 1A,B) in the initial phase. At this stage, the positive thyrotoxicosis induction rate was 78.9% (Table 1). Exogenous MLT treatment (MTG group) produced higher plasma T4 and T3 levels compared to the TG group, but without a significant change in the rate of positivity. In addition, there was no significant difference in T3 and T4 levels between the MLT alone group and the control group.

**Table 1:** Responses after human thyroglobulin immunization in different groups of CBA mice in the initial and recovery phases.

	Positive rate	Negative rate	*p* value
	N (%)	N (%)	
**Initial phase**			
TG	15 (78.9%)	4 (21.1%)	0.660
MTG	16 (88.9%)	2 (11.1%)	
**Recovery phase**			
TG	3 (42.9%)	4 (57.1%)	0.970
MTG	3 (42.9%)	4 (57.1%)	
TGM	3 (37.5%)	5 (62.5%)	

Positivity of thyrotoxicosis is defined as mice with a T4 level > mean + 2X standard deviations of the T4 level in control mice.

In the recovery phase, the positivity rates of thyrotoxicosis in the TG and MTG groups both declined to 42.9%, and only the MTG group showed a higher plasma T4 level than the control group (Fig. 1C,D). It was noted that treatment with MLT after the initial phase (TGM group) had no significant effects on plasma T4 and T3 levels or the positivity rate. In the present study, the average intake of MLT-containing water in each MLT-treated mouse was about 6.3 ml/day (≒37.8 μg/mice/day).

### Exogenous MLT showed limited effects on plasma ATA and TSHRAb levels with TG-induced thyroiditis

**Figure 2:**
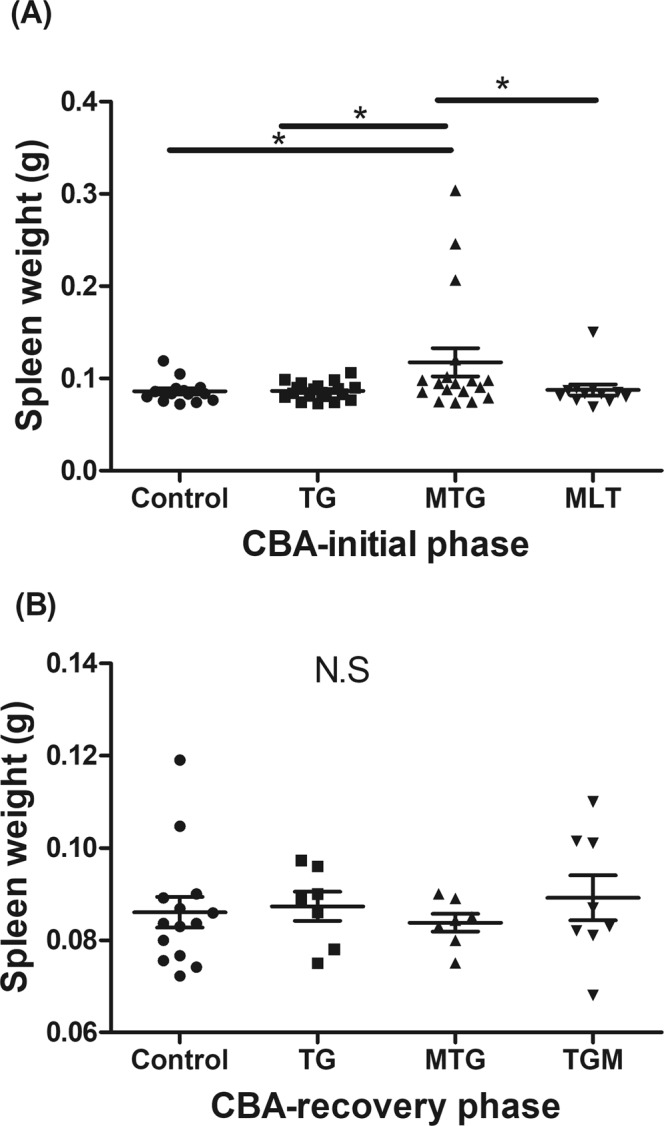
Anti-human thyroglobulin antibody titers in CBA mice in the initial (**A**) and recovery phases (**B**). Each bar represents the mean and standard error. *p < 0.05, **p < 0.01; ***p < 0.001.

In order to study the etiology of thyrotoxicosis in TG-induced thyroiditis, plasma TSHRAb titers and ATA levels were analyzed. As shown in Fig. 2, CBA mice in the TG and MTG groups had elevated ATA levels compared to those in the control and MLT groups in both the initial and recovery phases. However, there were no differences in ATA titers between the MTG and TG group in either phase (Fig. 2A,B). In addition, TSHRAb levels were not detected in all groups in either the initial or recovery phase (Supplementary Fig. 1A,B).

### Exogenous MLT treatment exacerbated follicle destruction and immune cell infiltration in TG-induced thyroiditis

**Figure 3:**
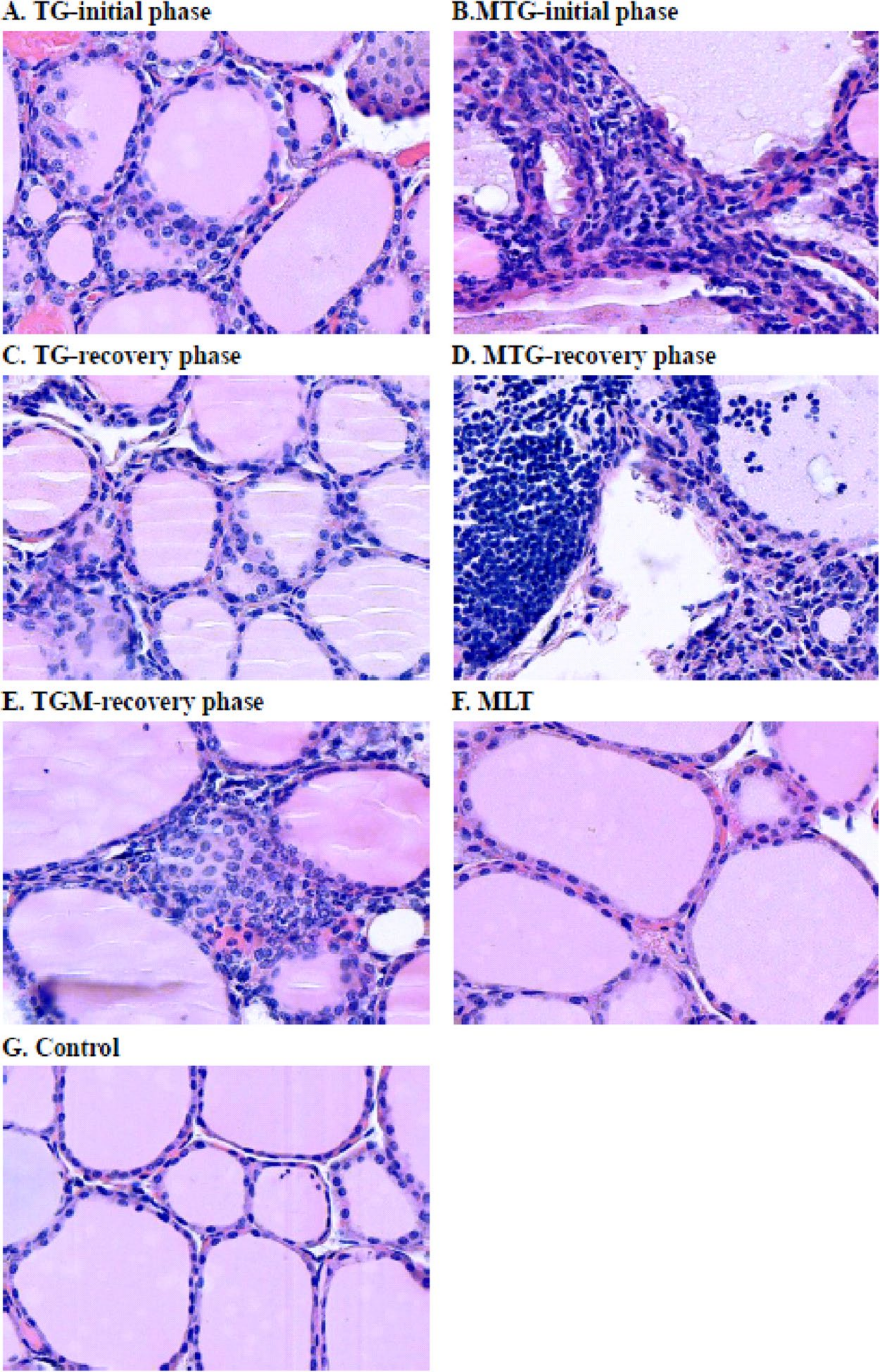
Histology of thyroid follicular cells in different groups of CBA mice. (**A**) TG-immunized mice showing mild thyroid follicle disruption and mononuclear cell infiltration in the initial phase (400x). (**B**) MTG group showing severe thyroid follicular destruction and profound mononuclear cell infiltration in the initial phase (400x). (**C**) TG-immunized mice showing mild thyroid follicle disruption and mononuclear cell infiltration in the recovery phase (400x). (**D**) MTG group showing severe thyroid follicular destruction and profound mononuclear cell infiltration in the recovery phase (400x). (**E**) TGM mice demonstrating mild to moderate thyroid follicle disruption and mononuclear cell infiltration in the recovery phase. (**F**) No thyroid follicle destruction observed in MLT-treated mice (400x). (**G**) Normal thyroid tissue of CBA mice (400x).

Gross pictures of the thyroid glands of the TG, MTG, MLT, and control groups in CBA mice are shown in Supplementary Fig. 2. There was no significant diversity in thyroid size among the four groups. Histological results of thyrocytes of the various groups in CBA mice in the initial and recovery phases are demonstrated in Fig. 3. In the initial phase, TG-immunized mice (Fig. 3A) showed mild thyroid follicle disruption together with minor mononuclear cell infiltration compared to the control group. Interestingly, we observed extensive thyroid follicle destruction and profound mononuclear cell infiltration in the MTG group (Fig. 3B) compared to the TG group. In the recovery phase, consistent thyroid follicle damage and increased infiltrating mononuclear cells in the MTG group were observed compared to the TG and TGM groups (Fig. 3C–E). In the meantime, neither thyroid follicle destruction nor increased infiltrating cells were observed in MLT-treated alone mice in the recovery phase compared to the control group (Fig. 3F,G).

### Exogenous MLT modulated TG-induced thyroiditis predominantly through T cell activation

**Figure 4:**
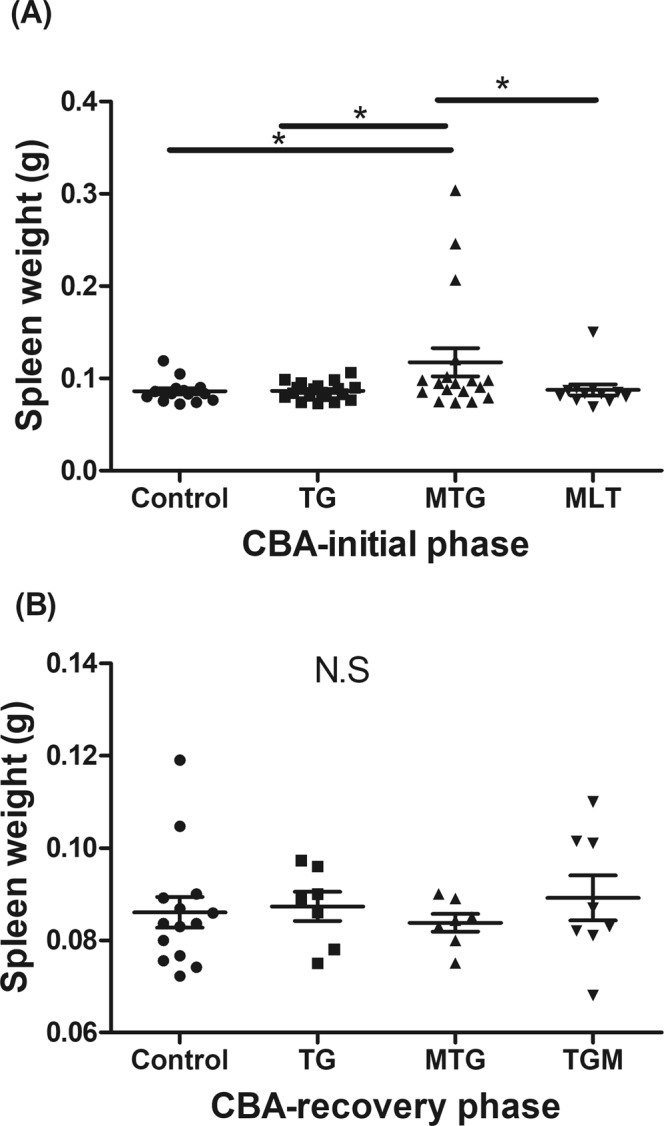
Spleen weights of CBA mice in the initial (**A**) and recovery phases (**B**). Each bar represents the mean and standard error. *p < 0.05; NS, non-significant.

**Figure 5:**
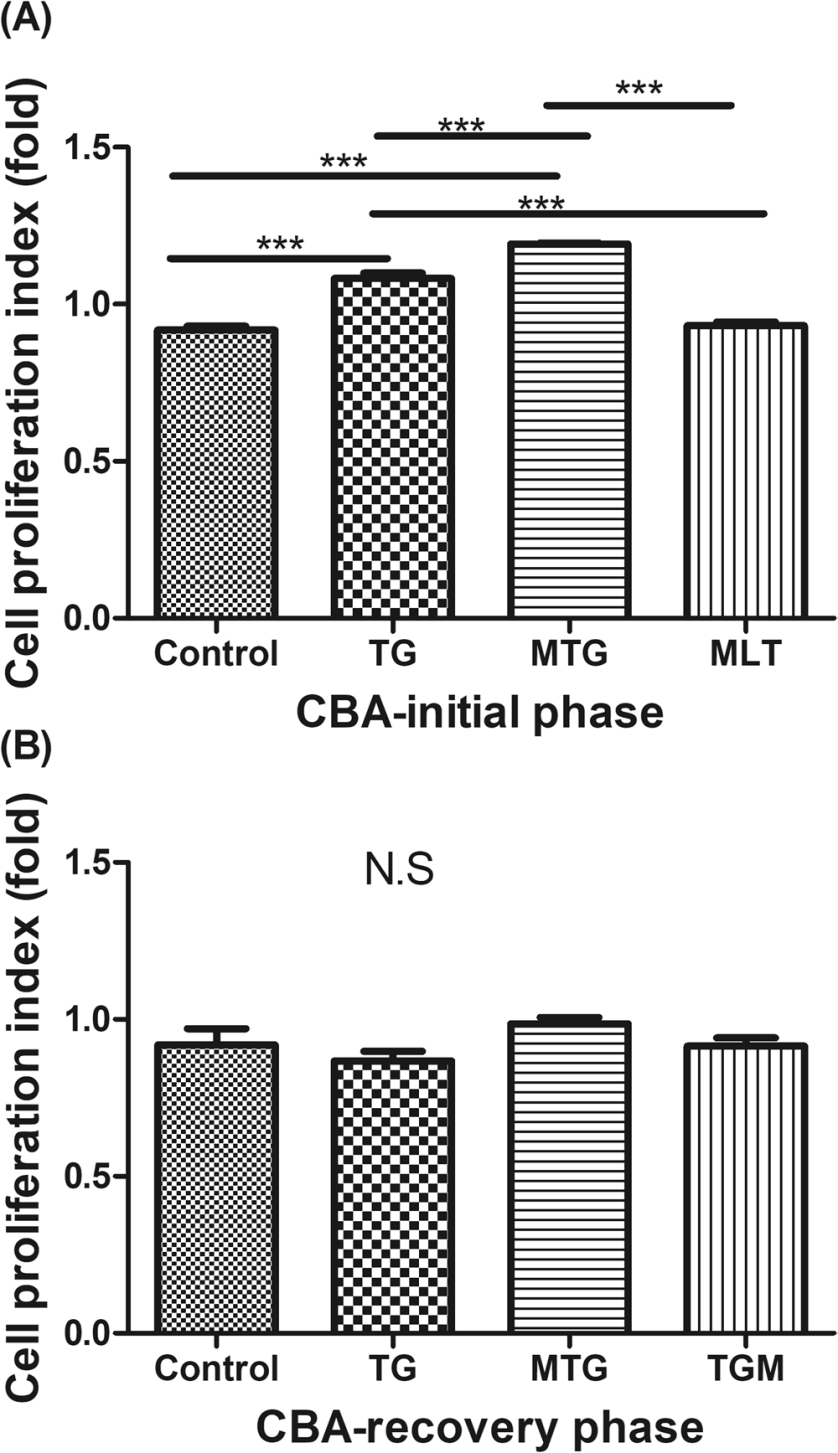
Cell proliferation index under stimulation with 10 μg/ml human thyroglobulin in CBA mice in the initial (**A**) and recovery phases (**B**). Each bar represents the mean and standard error. *p < 0.05, **p < 0.01; ***p < 0.001; NS, non-significant.

**Figure 6:**
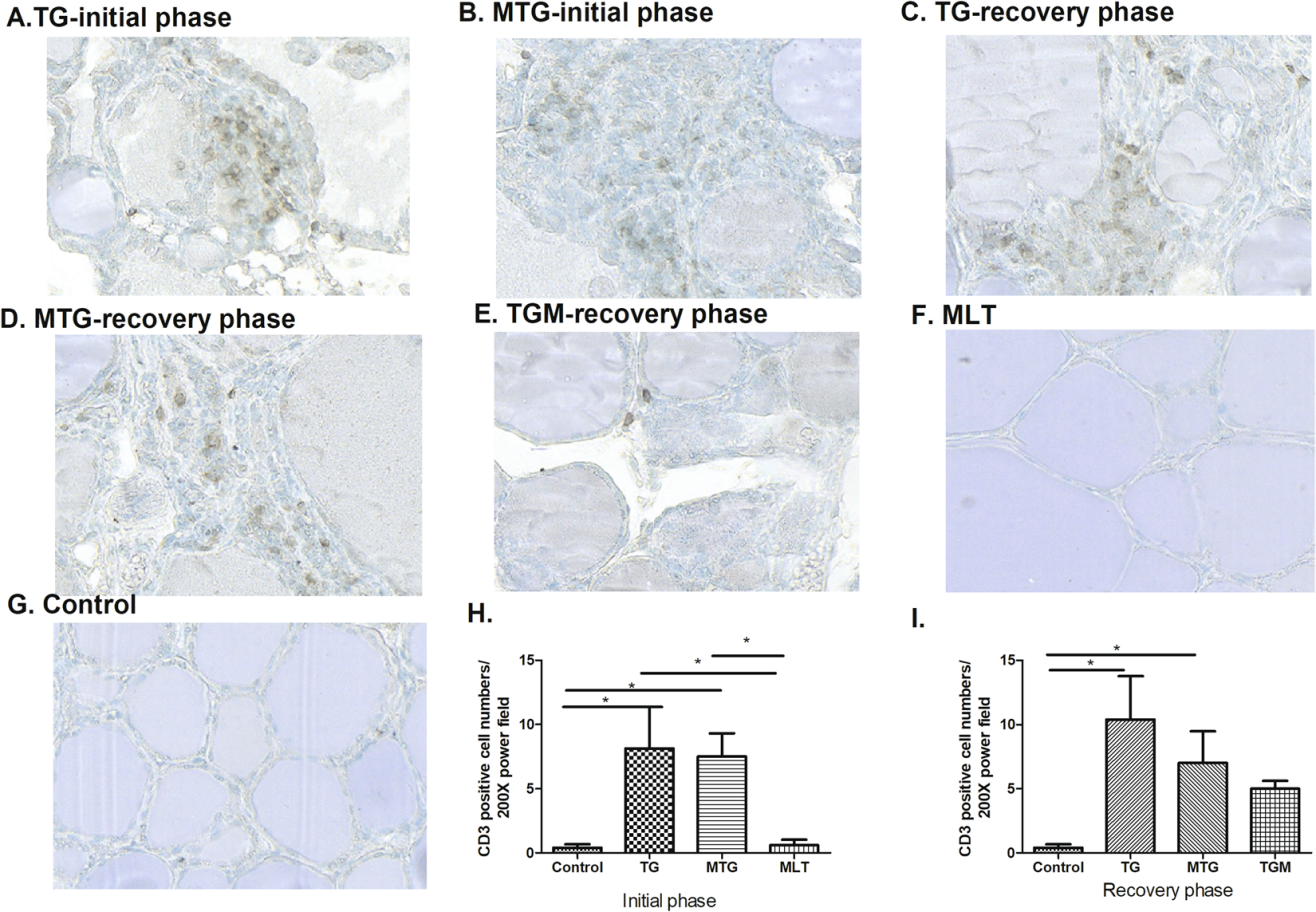
CD3 protein expressions in different groups of CBA mice. (**A**) TG-immunized mice showing focal CD3 protein expression in infiltrating cells in the initial phase (400x). (**B**) MTG group showing focal CD3 protein expression in infiltrating mononuclear cells in the initial phase (400x). (**C**) TG-immunized mice showing focal CD3 protein in infiltrating cells in the recovery phase. (**D**) MTG group showing focal interstitial CD3 protein expression in the recovery phase (400x). (**E**) TGM mice demonstrating few thyroid follicle disruptions and mononuclear cell infiltration in the recovery phase. (**F**) MLT-treated mice showing negative CD3 staining (400x). (**G**) Normal thyroid tissue of CBA mice showing negative CD3 staining (400x). The quantifications of the infiltrated CD3 positive cells in initial phase (**H**) and recovery phase (**I**). Each bar represents the mean and standard error. *p < 0.05.

The potential effects of MLT in modulating immunological features in TG-induced thyroiditis in CBA mice were further evaluated. In the initial phase, splenomegaly was only observed in the MTG group (Fig. 4A), and this phenomenon was reduced in the recovery phase (Fig. 4B). Splenocytes stimulated with TG had increased the cell proliferation index in the TG and MTG groups in the initial phase, but they had declined in the recovery phase (Fig. 5A,B). It was noted that the MTG group showed a higher cell proliferation index than the TG group (Fig. 5A). In addition, infiltration of CD3^+^ T cells was observed in the TG and MTG groups in both the initial and recovery phases (Fig. 6A–E), but not in the control or MLT-alone groups (Fig. 6F,G). The quantifications of the infiltrated CD3^+^ T cells of CBA mice were shown in Fig. 6(H,I). In initial phase, mice in TG and MTG groups had higher infiltrated CD3 + T cell numbers than MLT and control groups, while, there was no significant difference between the MLT and control groups and between TG and MTG groups (Fig. 6H). In the recovery phase, TG and MTG groups had higher infiltrated CD3^+^ T cell numbers than the control group, but there was no significant difference between the control and TGM groups. In addition, there was also no significant difference in infiltrated CD3 + T cell numbers among TG, MTG and TGM groups in recovery phase (Fig. 6I). This evidence indicated the detrimental role of infiltrating T cells in this TG-induced thyrotoxicosis mouse model; in addition, from the splenocyte stimulation assay, we observed that the MTG group exhibited a further increase in activation of T cells compared to the TG group.

### Biochemical results, gross pictures, and histology of thyroid glands in B6 mice

**Figure 7:**
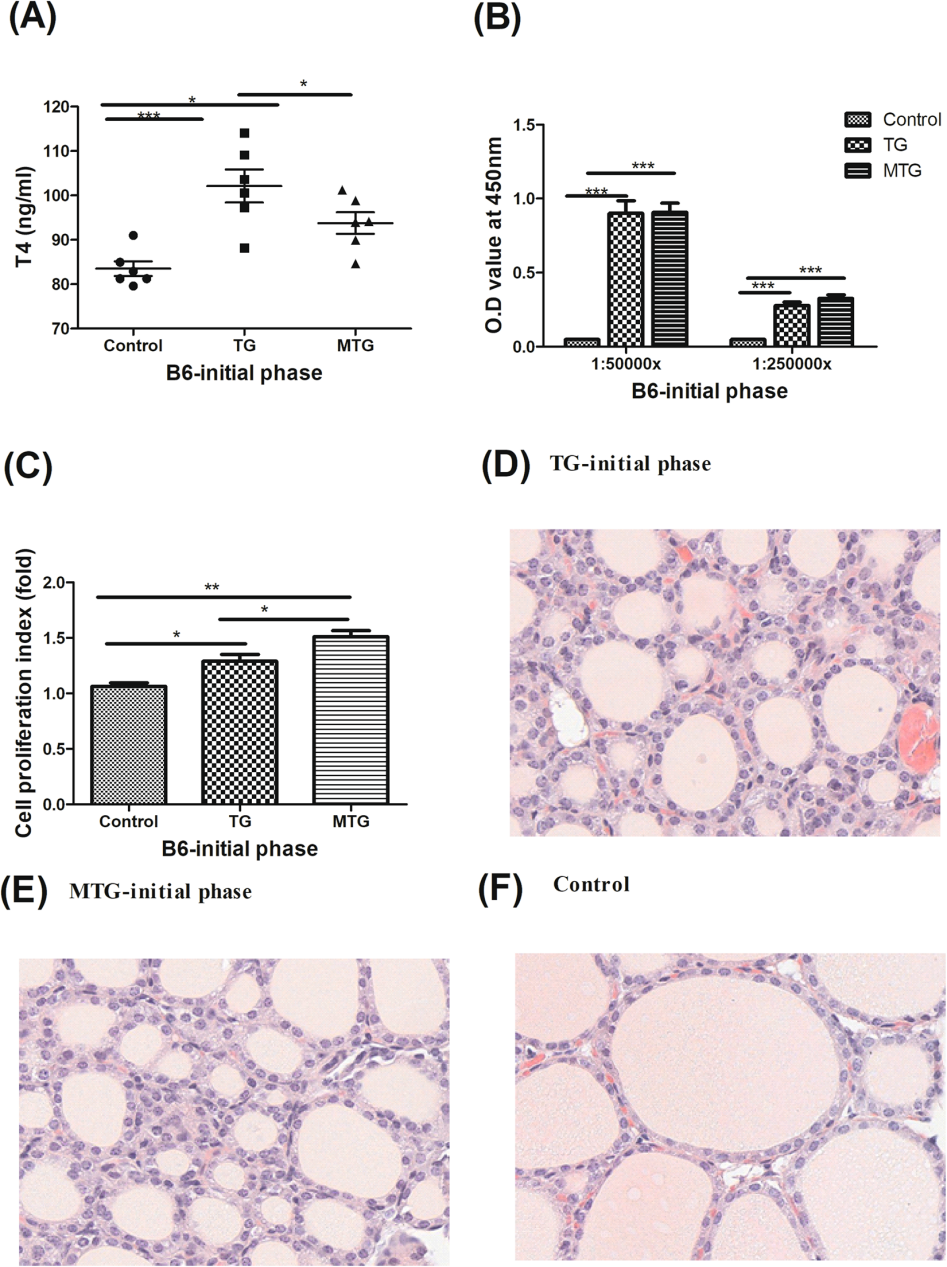
Thyroid functions (**A**), anti-human thyroglobulin antibody titer (**B**), cell proliferation index (**C**), and histology (**D**–**F**) in B6 mice. (**D**) TG-immunized mice showing mild thyroid follicle disruption and mononuclear cell infiltrations in the initial phase (400x). (**E**) MTG group showing mild thyroid follicular destruction and mononuclear cell infiltration in the initial phase (400x). (**F**) Normal thyroid tissue of B6 mice (400x). T4, thyroxine; Cell proliferation index, under stimulation with 10 μg/ml human thyroglobulin. Each bar represents the mean and standard error. *p < 0.05, **p < 0.01; ***p < 0.001.

It was reported that endogenous MLT is absent from B6 mice due to a lack of essential enzymes for MLT synthesis^21,22^. Therefore, B6 mice were also used to examine the effects of MLT in TG-induced thyroiditis. In the initial phase, B6 mice in both the TG and MTG groups had higher T4 levels than those in the control group (Fig. 7A), and the positivity of the thyrotoxicosis induction rate was 83.3% in both groups (Table 2). There were higher ATA levels in both the TG and MTG groups than in the control group, while there was no difference in ATA titers between the TG and MTG groups (Fig. 7B). In addition, no TSHRAb levels were detected in any group (Supplementary Fig. 1C). Splenocytes from both the TG and MTG groups had higher TG-induced cell proliferation indexes, and the MTG group had a higher cell proliferation index than that of the TG group (Fig. 7C). Thyroid sizes in the TG, MTG, and control groups of B6 mice are shown in Supplementary Fig. 2B, and no difference in thyroid sizes among the three groups was apparent. In the histological analysis, mice in the TG and MTG groups showed minor follicle disruption and increased mononuclear infiltration in thyroid histopathology compared to the control group (Fig. 7D–F). However, there was no difference in thyroid pathology observed between the TG and MTG groups of B6 mice.

**Table 2:** Responses after human thyroglobulin immunization in different groups of C57BL6 mice in the initial and recovery phases.

	Positive rate	Negative rate	*p* value
	N (%)	N (%)	
**Initial phase**			
TG	5 (83.3%)	1 (16.7%)	1.000
MTG	5 (83.3%)	1 (16.7%)	
**Recovery phase**			
TG	8 (100%)	0 (0%)	
MTG	8 (100%)	0 (0%)	
TGM	8 (100%)	0 (0%)	

Positivity is defined as mice with a T4 level of >mean + 2X standard deviations of the T4 level in control mice.

## Discussion

In this study, we created a mouse thyroiditis model by repeated TG injections in different mouse strains, which manifested as thyrotoxicosis, thyroid follicle destruction, and CD3^+^ T cell infiltration. At the same time, we also showed that exogenous MLT administration enhanced TG-induced thyroiditis in both the initial and recovery phases in CBA mice. In the meantime, MLT administration did not alter the serological or pathological features of thyroiditis in TG-treated B6 mice, which suggests that the influence of MLT in deteriorating thyroiditis is diverse in different strains. Moreover, MLT facilitated the splenocyte proliferation capacity in both strains rather than increased plasma ATA titers after TG immunization, which indicated that MLT exacerbated thyroiditis and thyrotoxicosis in TG-treated mice chiefly by way of T cell-mediated immunity, but not an antibody-driven immune reaction. Our results further confirmed the remarkable influence of the MLT signature in thyroid immune responses, and imply important clinical information that excess MLT intake may alter thyroid functions and aggravate thyroid autoimmunity in the development of AITD patients. Thus, caution should be exercised with the use of MLT in AITD susceptible population.

To clarify the role of MLT signals in AITD progression, we tried to build an EAT model by repeated TG injections according to a protocol proposed by Endo *et al*.^23^. It was well documented that EAT can be established in several mouse strains by injections of a mouse thyroid extract^24^. Interestingly, Valdutiu *et al*. first observed initial hypothyroidism at 2 weeks but elevated thyroid function at 4 weeks after the initial immunization in several mouse strains, which suggested a possible thyrotoxic phase after injecting mouse TG^25^. Recently, by repeated bovine TG injections in B6 and AKR mice, Endo *et al*. tried to create an EAT mouse model and found thyroid enlargement, thyroid follicular cell hyperplasia and hypertrophy, elevated thyroid function, the existence of the TSHRAb, and increased thyroid RAIU in week 12 after the first immunization, which was characterized by GD. They suggested that the pathogenesis of autoimmune thyroiditis and GD might be closely related, and the initial event of GD could be derived from autoimmune thyroiditis^23^. In the present study, we induced thyrotoxicosis in B6 and CBA mice. However, we were unable to demonstrate the presence of the TSHRAb in the bloodstream, and importantly, we observed thyrocyte destruction and increased infiltrating inflammatory mononuclear cells with CD3^+^, rather than thyrocyte hyperplasia and hypertrophy by histopathology in both strains, which strongly suggested autoimmune thyroiditis in essence. Moreover, in CBA mice, we observed a significant decline in plasma T4 levels from the initial phase to the recovery phase (*p* = 0.010, data not shown), which further implied that thyroid function in a TG-induced mouse model cannot be maintained like in a GD model. Finally, the actual mechanism of the conflicting results between ours and Endo *et al*. is unknown, and might be attributed to different adjuvant administration routes and sources of TG for immunization. In addition, the TSHR stimulating activity, measurement by cyclic AMP production as pervious work, was not determined in the present study^23^. It is also possible that some of these immunized mice with low TSHRAb titer might have high TSHR stimulating activity, and subsequently activate thyrocytes, and presented like GD. Further molecular-based studies of the detailed pathogenesis are needed.

In the present study, we further assessed the impacts of MLT administration on inducing and promoting disease progression in a TG-immunization mouse model. After MLT was given 1 week prior to the TG immunization, we found that there was no difference in the positive induction rates between the TG and MTG groups in the initial and late phases. However, we found deteriorated thyroid function and more-extensive thyrocyte destruction in the MLT treated TG-immunized CBA strain, and the facilitating effect of MLT in TG-immunized CBA mice was still present in the recovery phase according to the biochemical and histopathological analyses. At the same time, we also observed that MLT augmented the splenocyte TG-specific cell proliferation capacity, but not the plasma ATA titer, which suggested that initial MLT administration could facilitate thyrotoxicosis chiefly by operating the T cell-mediated immune reaction rather than antibody-driven immunity in the early stage in the TG-immunized CBA strain. Our results were compatible with observations from other reports, which demonstrated that chronic MLT administration can enhance Th1 cytokine production and promote Th1 immunity polarization^4,26–28^. Our results were also in line with the findings conducted by Baltaci *et al*., which showed that MLT could play a crucial role in the cell-mediated immunity *in vivo*^29,30^. In contrast, MLT administration after completing four doses of TG immunization did not alter thyroid function, ATA titers, splenocyte cell proliferation capacities, or thyroid histopathology compared to those of the TG-immunized alone group of CBA mice, which indicated that late MLT administration might not significantly contribute to the regulation of thyroid function or thyroid immunity in a full-blown TG-immunized CBA mouse model. This evidence suggests that MLT contributed to the process of the progression of thyroiditis, but showed slighter effects on modifying the established disease status. Interestingly, we also found that there was no significant change in thyroid function (T3, T4), thyroid histopathology, or the splenocyte cell proliferation index between the MLT and control groups of CBA mice, which suggested that MLT alone might play a very limited role in thyroid function and the induction of de novo thyroid autoimmunity in the normal thyroid.

Interestingly, the influence of enhancing thyrotoxicosis and thyrocyte damage by MLT administration was not observed in B6 mice, while the MLT-promoting TG-specific T-cell proliferation ability was preserved. These findings imply that MLT exhibited a minor effect on altering thyroid autoimmunity in TG-immunized B6 mice compared to TG-immunized CBA mice. Variations in thyroid function between CBA and B6 mice may be attributed to distinct strain-specific immune reactions to the antigen or immunoregulator. Moreover, CBA mice are known to be able to synthesize MLT, while MLT is absent from B6 mice due to a lack of essential enzymes for MLT synthesis. Exogenous MLT administration could lead to excessive MLT accumulation in CBA mice, but not in the B6 strain, which might further contribute to the profound thyroid dysfunction and immune dysregulation in TG-immunized CBA mice compared to B6 mice^21,22^. In other words, higher dose of MLT might be required to demonstrate comparable effect in B6 mice than in CBA mice.

To our best knowledge, this is the first study to show that MLT administration increases plasma thyroid hormone levels and enhances thyrocyte destruction and the T-cell proliferation capacity in a TG-immunized thyroiditis mouse model. The combined results of the animal and our previous genetic-association studies highlight how the MLT signaling pathway plays a considerable role in the development and modulation of AITD^20^. However, we should point out certain limitations of the study. First, the RAIU of the mice thyroid gland were not quantified in the study, which could have further clarified the presence of thyroiditis in the mice model. Second, the daily exogenous MLT uptake on average was about 37.8 μg/mice in the present study. The results might differ with other concentrations of MLT treatment; further well-designed studies with different dosages of MLT to explore the responses of MLT in HT models are required. Finally, compared to the “typical form of HT”, the thyrotoxic phase of thyroiditis as in the present study is less frequently seen in clinical settings. Our future plan is to create a classical HT model to clarify the role of MLT in the progression of HT.

In conclusion, the presence of increasing MLT enhanced the T cell immune response and early exogenous MLT administration exacerbated the clinical and pathological features of TG-induced thyroiditis.

## Materials and Methods

### Immunization of mice

Briefly, 42 female B6 and 85 CBA mice were purchased from the National Laboratory Animal Breeding and Research Center (Taipei, Taiwan). All mice were 12~14 weeks old at the beginning of the experiments. TG, purchased from Calbiochem (La Jolla, CA), was emulsified with the same volume of complete Freund’s adjuvant (CFA, Wako Chemical, Tokyo, Japan) and then 50 μl of an emulsion (25 μg TG/mouse) was injected into the soleus muscle of each mouse at week 0. Two weeks later, TG was emulsified with the same volume of incomplete Freund’s adjuvant (ICFA, Wako Chemical) and was injected once every 2 weeks with a total of three doses (at weeks 2, 4, and 6). Saline emulsified with the same volume of either CFA (at week 0) or ICFA (at week 2, 4, and 6) was injected into the control group.

**Figure 8:**
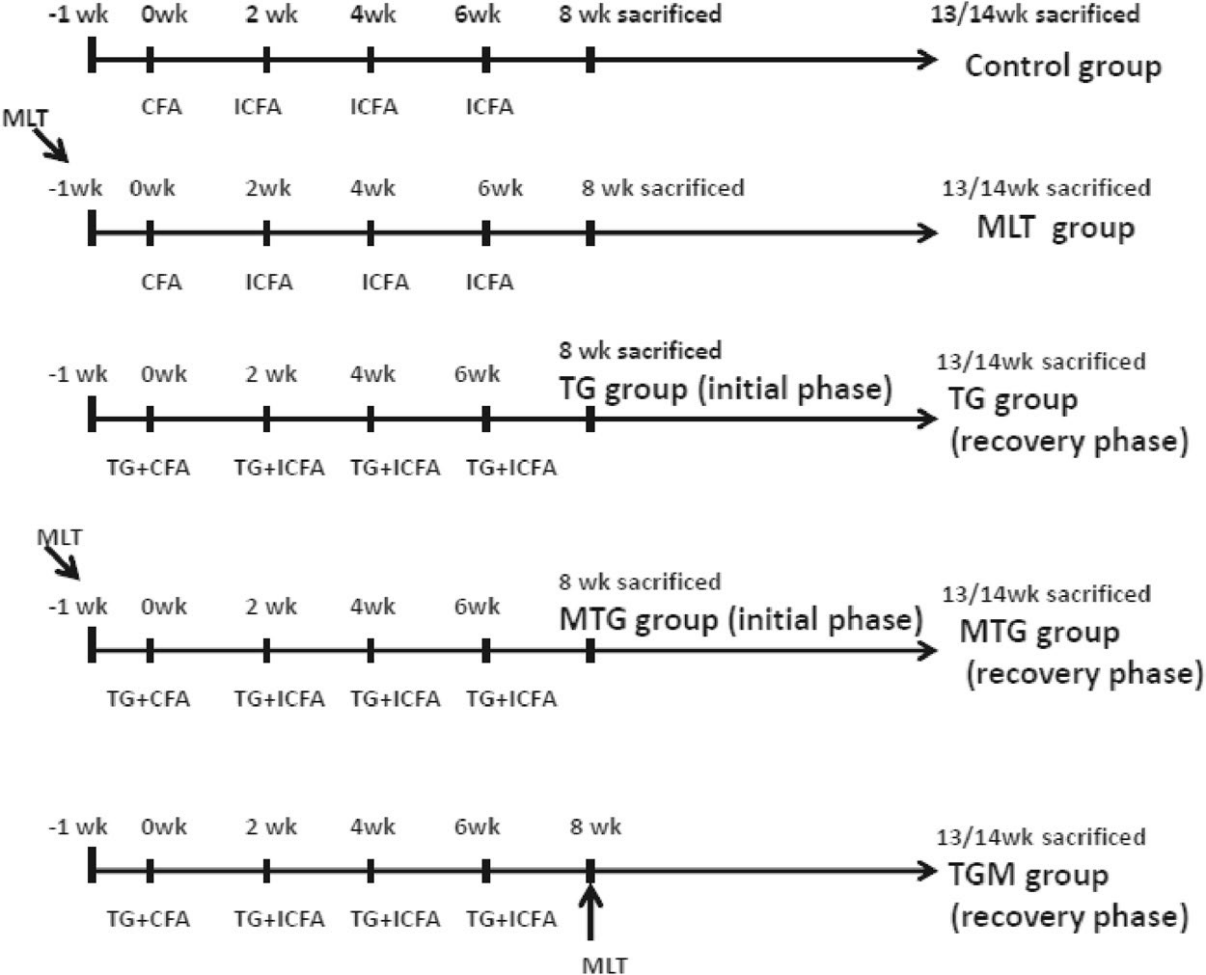
The scheme of immunization and treatment protocol CFA, complete Freund’s adjuvant; ICFA, incomplete Freund’s adjuvant.

The different treatment groups were defined as follows: TG group, TG immunization without MLT treatment; MTG group: TG immunization with MLT treatment, MLT administration 1 week before the first TG immunization; MLT group: treated as the control group, but MLT was administered 1 week before the first CFA injection; TGM, MLT administration began in the initial phase. MLT (Sigma-Aldrich, St. Louis, MO, USA) was dissolved in daily drinking water at a concentration of 6 μg/ml in a water bottle protected against light in every cage^31^. The daily water intake in each group did not differ significantly during entire course of the experiment. Mice were sacrificed at either 8 (initial phase) or 13~14 weeks (recovery phase). The immunizations and treatments in each group were shown in Fig. 8. The protocol and procedures employed were ethically reviewed and approved by the Institutional Animal Care and Use Committee of Taipei Medical University and all methods were performed in accordance with the relevant guidelines and regulations.

### Total T4 and Total T3 measurements (enzyme-linked immunosorbent assay (ELISA))

The microplate provided in the ELISA kit was pre-coated with an antibody specific to T4 or T3 (CUSABIO Biotech, Wuhan, China). A standard or sample was added to the appropriate microtiter plate wells with biotin-conjugated T4/T3. A competitive inhibition reaction was launched between T4/T3 and biotin-conjugated T4/T3 with the pre-coated antibody specific to T4/T3. After washing, avidin-conjugated horseradish peroxidase was added to the wells. A substrate solution was added to the wells, and the color was allowed to develop in contrast to the amount of T4/T3 in the sample. Color development was stopped, and the intensity of the color was measured at OD 450 nm.

### ATA measurement

Plasma samples from each group were collected at 8 weeks after the first TG immunization. ELISA plates were coated with TG (1 mg/ml in carbonate buffer) and blocked with 5% bovine serum albumin (BSA). Following extensive washing, mouse sera [diluted 1:200 in Tris-buffered saline] was added to the wells. Bound antibodies were detected using anti-mouse immunoglobulin G (IgG) rabbit serum conjugated to alkaline phosphatase (Jackson Immuno-Research, West Grove, PA), and the intensity of the color in the plates was measured at OD 450 nm.

### Thyroid-stimulating hormone (TSH) receptor (TSHR) antibody (TSHRAb)

Serum TSHRAb concentrations were quantified by an ELISA method using a commercial TSH receptor-coated plate (RSR Ltd, Cardiff, UK). A competitive inhibition reaction was initiated between the TSHRAb and M22-peroxidase with the pre-coated TSHR. The greater the amount of the TSHRAb in a sample, the less antibody that was bound by the biotin-conjugated TSHRAb. The tetramethylbenzidine (TMB) solution was added to the wells, and the color developed in contrast to the concentration of the TSHRAb in the sample. Color formation was discontinued, and the intensity of the color was measured at OD 450 nm on an ELISA reader. The results of individual samples were obtained after the negative control was assigned to a value of 0.1 μg/L, and a standard curve was created^32^.

### Immunohistochemistry (IHC)

IHC staining of thyroid tissues was performed using labeled streptavidin-biotin (LSAB 2 kit; DAKO, Carpentaria, CA, USA), as previously described^33,34^. Briefly, 3-µm paraffin sections were prepared on silane-coated microslides and deparaffinized in a xylene solution (Paraclear; Earthsafe Tech, Belle Mead, NJ) for 15 min. After rehydration in a graded (95%, 80%, and 75%) alcohol series, sections were microwaved in 0.01 M sodium citrate buffer for 15 min. Sections were blocked using TBST containing 2% BSA for 30 min and then incubated at 4 °C overnight with a 1:100 dilution of CD3 (GTC16667, Genetax, San Antonio, TX). After washing, sections were incubated with an anti-goat IgG-biotin conjugate (Santa Cruz Biotechnology, Santa Cruz, CA) for 60 min, followed by streptavidin-horseradish peroxidase (EnVision DAB kit, DAKO) for 20 min. To determine peroxidase activity, sections were incubated with 3,3-diaminobenzidine (DAB, DAKO) for 10 min in the dark. Finally, sections were counterstained with hematoxylin-eosin for histological examination. The average numbers of infiltrated CD3 positive cell were quantified from five randomly non-overlapping 200X power field in each tissue samples.

### Splenocyte proliferative assay

The spleens of immunized mice were meshed into single cell suspensions and cultured in RPMI medium containing 5% fetal bovine serum (FBS). Splenocytes were seeded at 2 × 10^6^ cells/ml and 200 μl/well in a 96-well flat-bottom plate and stimulated with 0, 1, and 10 μg/ml of TG. Cell proliferation was further assessed by adding a mixture of the tetrazolium salt, 3-(4,5-dimethylthiazol-2-yl)-5-(3-carboxymethoxyphenyl)-2-(4-sulfophenyl)-2H-tetrazolium (MTS) and phenazine methosulphate, and the intensity of the color in the plates was measured at OD 490 nm.

### Statistical analysis

SPSS vers. 13.0 for Windows (SPSS, Chicago, IL, USA) was used for all statistical analyses. Quantitative values are shown as the mean ± standard error (SE). A one-way analysis of variance (ANOVA) was used to compare differences in clinical parameters among the different groups. The LSD test was used for post-hoc examinations. The positivity of thyrotoxicosis induction was defined as previously described^35^, mouse with a plasma T4 value greater than the mean + 2 X standard deviations of T4 obtained from the control group. All statistical tests were two-sided, and a *p* value of < 0.05 was considered significant.

## Supplementary information

Supplementary Figures
