# Author Correction: Effects of exogenous melatonin on clinical and pathological features of a human thyroglobulin-induced experimental autoimmune thyroiditis mouse model

**DOI:** 10.1038/s41598-019-44363-4

**Published:** 2019-06-06

**Authors:** Jiunn-Diann Lin, Wen-Fang Fang, Kam-Tsun Tang, Chao-Wen Cheng

**Affiliations:** 1 Graduate Institute of Clinical Medicine, College of Medicine, Taipei Medical University, Taipei, Taiwan; 2 Division of Endocrinology, Department of Internal Medicine, Shuang Ho Hospital, Taipei Medical University, New Taipei City, Taiwan; 3 Division of Endocrinology and Metabolism, Department of Internal Medicine, School of Medicine, College of Medicine, Taipei Medical University, Taipei, Taiwan; 4 Department of Family Medicine, Shuang Ho Hospital, New Taipei City, Taiwan; 5 Division of Endocrinology and Metabolism, Department of Internal Medicine, Veterans General Hospital, Taipei, Taiwan; 6 Traditional Herb Medicine Research Center, Taipei Medical University Hospital, Taipei Medical University, Taipei, Taiwan; 7 Cell Physiology and Molecular Image Research Center, Wan Fang Hospital, Taipei Medical University, Taipei, Taiwan

Correction to: *Scientific Reports* 10.1038/s41598-019-42442-0, published online 10 April 2019

This Article contains errors.

As a result of a mistake in preparation of the final version of the manuscript, Figure 2 in the Article duplicates Figure 4. The correct version of Figure 2 has been included with the paper throughout the review process and is now included below as Figure 1.

**Figure 1:**
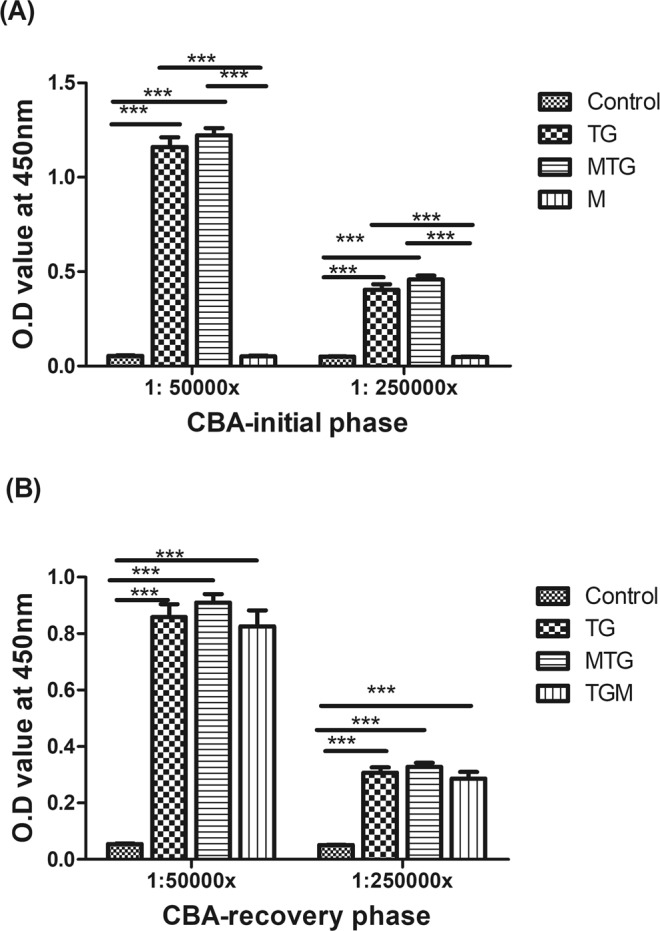
Anti-human thyroglobulin antibody titers in CBA mice in the initial (**A**) and recovery phases (**B**). Each bar represents the mean and standard error. *p < 0.05, **p < 0.01; ***p < 0.001.

The Authors apologise for the error.

