# Supplementary material for: Effects of exogenous melatonin on clinical and pathological features of a human thyroglobulin-induced experimental autoimmune thyroiditis mouse model
Source: Sci Rep. 2019 Apr 10;9:5886. doi: 10.1038/s41598-019-42442-0 (PMC6458129; doi:10.1038/s41598-019-42442-0)
Supplement: Supplementary file 1 — Supplementary Figures [file 41598_2019_42442_MOESM1_ESM.docx]

Effects of exogenous melatonin on clinical and pathological features of a human thyroglobulin-induced experimental autoimmune thyroiditis mouse model

# Jiunn-Diann Lin^1,2,3^, Wen-Fang Fang^4^, Kam-Tsun Tang^5^, Chao-Wen Cheng^1, 6*^

^1^Graduate Institute of Clinical Medicine, College of Medicine, Taipei Medical University, Taipei, Taiwan; ^2^Division of Endocrinology, Department of Internal Medicine, Shuang Ho Hospital, Taipei Medical University, New Taipei City, Taiwan; ^3^Division of Endocrinology and Metabolism, Department of Internal Medicine, School of Medicine, College of Medicine, Taipei Medical University, Taipei, Taiwan; ^4^Department of Family Medicine, Shuang Ho Hospital, New Taipei City, Taiwan; ^5^Division of Endocrinology and Metabolism, Department of Internal Medicine, Veterans General Hospital, Taipei, Taiwan;^6^Traditional Herb Medicine Research Center, Taipei Medical University Hospital, Taipei Medical University, Taipei, Taiwan.

Run title: Melatonin in AITDs

Address correspondence to:

^*^Chao-Wen Cheng, PhD, Graduate Institute of Clinical Medicine, College of Medicine, Taipei Medical University, 250 Wuxing St., Taipei 11031, Taiwan, ROC. E-mail: ccheng@tmu.edu.tw

Supplemental Figure 1. Thyroid-stimulating hormone receptor antibody (TSHRAb) in different groups of CBA mice in the initial and recovery phases and B6 mice in the initial phase.


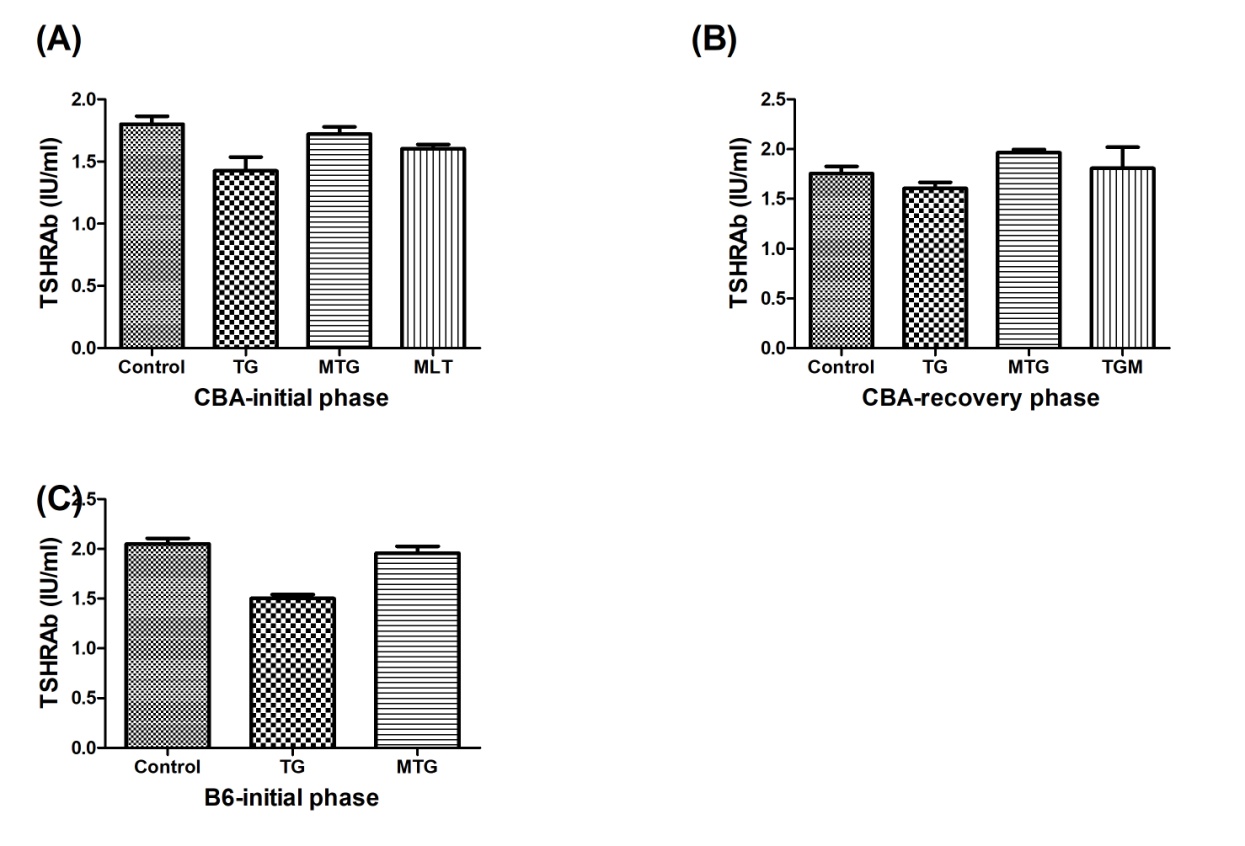


Each bar represents the mean and standard error. * *p*<0.05, ** *p*<0.01; *** *p*<0.001.

Supplemental Figure 2. The gross appearance of the thyroid in each group: (A) CBA mice; (B) B6 mice in the initial phase.

| A.   |
| --- |
| B.   |
